# Dental care utilization: examining the associations between health services deficits and not having a dental visit in past 12 months

**DOI:** 10.1186/s12889-019-6590-y

**Published:** 2019-03-05

**Authors:** M. Nawal Lutfiyya, Andrew J. Gross, Burke Soffe, Martin S. Lipsky

**Affiliations:** 1 Department of Pharmacy Practice and Pharmaceutical Sciences, College of Pharmacy, University of Minnesota, Minneapolis, MN USA; 2 College of Dental Medicine, Roseman University of Health Sciences, 10920 S. River Front Parkway, South Jordan, Utah 84095 USA

## Abstract

**Background:**

A growing literature supports the contention that closing the divide between dental and medical care can improve access to and coordination of patient care. Health service deficits (HSDs) entail: no routine medical exam, no personal healthcare provider (HCP), no health insurance, and/or delaying medical care because of cost all within the last 12 months. Examining the associations between HSDs and dental care utilization could inform strategies and interventions aimed at narrowing the gap between the medical and dental professions. This study explored whether HSDs are associated with not having a dental care visit within the last 12 months. In addition, the study sought to provide an updated analysis of the characteristics and factors associated with dental care utilization.

**Methods:**

Two thousand sixteen Behavioral Risk Factor Surveillance System survey data were analyzed using bivariate and multivariable techniques. The outcome variable for this study was: *last dental visit was longer than 12 months ago*.

**Results:**

US adults without healthcare insurance, without a personal HCP, who had delayed medical care because of cost, and who had their last routine medical visit longer than 12 months ago had greater odds of not having a dental visit within the last 12 months. Further, this study identified disparities in dental care utilization among males, rural residents, those earning less than $50,000 per year, Non-Hispanic Blacks and Non-Hispanic other races. Individuals with six or more and/or all of their permanent teeth removed and current smokers also had greater odds of not having had a dental care visit in the past 12 months.

**Conclusions:**

Findings suggest that a stronger integration of medical and dental care might increase dental care utilization. In addition, persistent disparities in dental care utilization remain for several demographic groups. Targeted interventions offer the promise of helping achieve HP 2020 goals for improved oral health.

## Background

In the United States (US) there has been a long standing separation between the dental and medical professions [1–4]. This divide persists despite: 1) recognition of the importance of oral health to the physical and social well-being of individuals and overall population health [5], and 2) burgeoning arguments that the integration of dental and primary care might improve access to both [1–4]. There is a growing realization that comprehensive healthcare requires that the mouth needs to be put back into the body [1–4] and that oral health affects far more than teeth and a person’s smile [6–14]. Bad dentition can lead to social stigmatization, poor self-esteem and negatively affect an individual’s quality of life [9–11]. Good oral health is also acknowledged as a crucial contributory factor to an individual’s overall health status [15]. Research demonstrates a connection between oral health and systemic illnesses including cardiovascular disease [6, 7], premature or low birth weight babies [16], depression [17, 18], asthma [19] and chronic obstructive pulmonary disease [20].

A growing literature [1–4] presents the case for reducing the divide between the practice of medicine and dentistry. Atchison and colleagues observed [3], that by integrating dental and medical care, access to and coordination of patient care is improved. A significant dimension of this integration entails interprofessional collaboration, an approach to healthcare delivery supported by the World Health Organization (WHO) [2]. Interprofessional collaboration occurs when healthcare personnel from different professions (such as medicine and dentistry) work together to provide comprehensive healthcare and related services to patients and communities [21].

Two leading Healthy People 2020 (HP2020) oral health indicators address the importance of receiving dental care as well as the importance of integrating dental and primary care [22]. These objectives are:

- Increase the proportions of children, adolescents, and adults who used the oral health care system in the past year with the target set at 49%.
- Increase the proportion of local health departments and Federally Qualified Health Centers (FQHCs) that have an oral health program.

Increasing dental visits serves to identify and to treat existing dental disease as well as providing opportunities for preventive care and for identifying oral manifestations of systemic disease [23–26]. Supporting interprofessional care practice and collaboration by enhancing the US public health structure and FQHC network is one example that offers the potential of expanding dental care access.

Studies examining dental care utilization and HP2020 goals, also highlight the importance of understanding factors affecting dental care utilization [23–40]. Recent changes in the healthcare landscape influencing primary medical care may have also impacted dental care utilization. For example, the 2010 Affordable Care Act (ACA) [41] expanded Medicaid coverage for adults and one study attributed a 3 to 6% in dental care utilization to the ACA with the greatest impact on low income adults [31]. By mandating oral healthcare coverage for children up to age 21, the ACA might motivate adults without coverage to seek care for themselves if their children made regular dental visits.

Health service deficits (HSDs) [42–45] are an evolving concept that assess healthcare access and utilization. Conceptually healthcare access and utilization are distinct yet inter-related since access is necessary for utilization. According to the Institute of Medicine (IOM) access to health services entails “the timely use of personal health services to achieve the best health outcomes.” [46] Access involves being able to make use of necessary healthcare (often through health insurance coverage), at a physical location (geographic availability), from a healthcare provider that the patient is able to communicate with and trusts (personal relationship) [47]. Healthcare utilization, in contrast, entails the actual use of healthcare services (e.g., mammograms, physical exams). Healthcare is accessed and used for a multiplicity of reasons such as preventing and treating health problems [48].

HSDs, as defined in this and other studies [42–45], include the factors of no routine medical exam in the past 12 months, not having a personal healthcare provider in the past 12 months, not having health insurance in the past 12 months, and having delayed medical care in the past 12 months because of cost. These four factors represent HSDs in part because of their direct (although not comprehensive) connection to preventive healthcare utilization and/or access to healthcare. At present, in the US, having health insurance and/or having the financial means for out-of-pocket costs often determines one’s access to healthcare. The two factors of annual or routine medical check-ups (utilization) and/or having a personal healthcare provider (access) are essential for the receipt of preventive healthcare in the US.

The potential impact of HSDs is increasingly important in the social context of the changing landscape of healthcare. If US adults who haven’t visited a dental professional in the past 12 months have additional HSDs, then integrating dental and primary care *may be* a key first step in expanding access to comprehensive care. Specifically, this study sought to investigate the question of whether HSDs are associated with US adults not having a dental care visit within the last 12 months. Examining the associations, if any, between HSDs and dental care utilization should inform health and dental care planners seeking to narrow the gap between the medical and dental professions with the goal of improving overall health. We hypothesize that after controlling for confounding covariates, HSDs are independently associated with not having had a dental visit in the past 12 months.

In addition to answering the research question posed, this study also provides an updated analysis of the characteristics and factors associated with dental utilization and identified risk factors associated with not visiting a dentist in the past 12 months. Exploring risk factors for underutilization provides insight into developing targeted strategies to address disparities in oral health [49]. This analysis is timely since a full assessment of HP2020’s goal to achieve health equity is approaching. Implicit in HP2020’s focus on health equity is an understanding of the foundational principles of social epidemiology that materially shape the way that diseases are experienced [50]. Societal factors continuously evolve and require periodic reinvestigation of risk factor patterns in order to develop interventions sensitive to changes in the relationship between health conditions and social factors [51].

## Methods

This study analyzed 2016 Behavioral Risk Factor Surveillance System survey (BRFSS) data to answer the research question and to test the study hypothesis. Bivariate and multivariable techniques were used. Two thousand sixteen data were analyzed because these represent the most recently available BRFSS data set.

As a random digit telephone survey, BRFSS is a collaborative project between the CDC and all US states and territories. The survey is administered to the non-institutionalized US adult population aged 18 years and older. BRFSS uses a complex multi-stage sampling approach and develops a weighting factor for application to the data in order to ensure that they are representative of the non-institutionalized US population based on the most recent census data. A more in-depth description of the data weighting process can be found elsewhere [52].

BRFSS collects information from individuals on health risk behaviors, preventive health practices, chronic conditions, and healthcare access primarily related to chronic disease and injury. Further, the survey collects data on a number of demographic and health services variables. The survey is composed of core questions that must be asked of every survey participant, as well as optional modules that may be chosen by individual states and asked only of the survey respondents from those participating state(s). Two core questions addressing oral health are asked of all survey respondents. These are:

1. How long has it been since you last visited a dentist or a dental clinic for any reason? Include visits to dental specialists, such as orthodontists.
2. How many of your permanent teeth have been removed because of tooth decay or gum disease? Include teeth lost to infection, but do not include teeth lost for other reasons, such as injury or orthodontics. (If wisdom teeth are removed because of tooth decay or gum disease, they should be included in the count for lost teeth)

The dependent variable for this study was derived from responses to the first question on oral health and was: *last dental visit was longer than 12 months ago*. Responses to the second question comprised one of the independent variables--- number of permanent teeth removed. In addition, this study included 17 independent variables. The independent variables included in this study included six demographic variables (sex, age, marital status, geographic locale, race/ethnicity, and veteran status), three socio-economic variables (education attained, employment status, and annual household income), four health service variables (have a health care provider, delayed medical care because of cost, timing of last routine medical check-up, and health insurance status), three health variables (self-defined health status, number of permanent teeth removed, and smoking status), and one access to healthcare variable (dentists per 100,000 population in state of residence). All but one of the independent variables came from BRFSS. Active Dentists Per 100,000 Population [50], a state level variable, was merged with the 2016 BRFSS data.

For analysis, all variables (except sex) underwent re-coding. Re-coding for the most part entailed collapsing response categories and removing the response categories of don’t know and refused. The categories of don’t know and refused for all variables included in the study were treated as missing and removed from the analysis. All variable re-codes were undertaken for clarity of the factors used for analysis and ease of interpretation. Table 1 displays the study variables by their original factors and re-coded factors.

**Table 1:** Study Variables Original and Re-coded Factors

Variable Type	Study Variables	Original Factors	Re-coded Factors for Analysis
Dependent Variable	Last Dentist Visit^a^	Within the past year	Within Last 12 Months^b^
		Within the past 2 years	Longer Than 12 Months Ago
		Within the past 5 years	
		5 or more years ago	
		Never	
		Don’t know/Not sure	Removed for analysis
		Refused	
		Missing	
Demographic Variables	Sex	Male	Male
		Female	Female
	Age	Age in years (18 to 99)	18–44 Years
			45–64 Years
			65 Years And Older
	Marital Status	Married	Married
		Divorced	Not Married
		Widowed	
		Separated	
		Never married	
		A member of an unmarried couple	Partner In An Unmarried Couple
		Refused	Removed for analysis
	Geographic Locale	In the center city of an MSA	Metropolitan
		Outside the center city of an MSA but inside the county containing the center city	
		Inside a suburban county of an MSA	
		Not in an MSA	Non-Metropolitan
	Race/Ethnicity	White only, non-Hispanic	White Non-Hispanic
		Black only, non-Hispanic	Black Non-Hispanic
		American Indian or Alaskan Native only, Non-Hispanic	Other Non-Hispanic
		Asian only, non-Hispanic	
		Native Hawaiian or other Pacific Islander only, Non-Hispanic	
		Other race only, non-Hispanic	
		Multiracial, non-Hispanic	
		Hispanic	Hispanic
		Don’t know/Not sure/Refused	Removed for analysis
	Veteran Status	Yes	Veteran
		No	Not A Veteran
		Don’t know/Not Sure	
		Refused	Removed for analysis
Socio-economic Variables	Education Attained	Never attended school or only kindergarten	Less Than High School
		Grades 1 through 8 (Elementary)	
		Grades 9 through 11 (Some high school)	
		Grade 12 or GED (High school graduate)	High School Graduate
		College 1 year to 3 years (Some college or technical school)	
		College 4 years or more (College graduate)	University Graduate
		Refused	Removed for analysis
	Employment Status	Employed for wages	Employed
		Self-employed	
		Out of work for 1 year or more	Not Employed
		Out of work for less than 1 year	
		A homemaker	Not Working By Choice Or Unable To Work
		A student	
		Retired	
		Unable to work	
		Refused	Removed for analysis
	Annual Household Income	Less than $10,000	Less Than $50,000
		$10,000 to less than $15,000	
		$15,000 to less than $20,000	
		$20,000 to less than $25,000	
		$25,000 to less than $35,000	
		$35,000 to less than $50,000	
		$50,000 to less than $75,000	$50,000 And More
		$75,000 or more	
		Don’t know/Not sure	Removed for analysis
		Refused	
Health Services Variables	Health Insurance Status	Yes	Have Health Insurance
		No	Do Not Have Health Insurance
		Don’t know/Not Sure	
		Refused	Removed for analysis
	Personal HCP	Yes, only one	Have HCP
		More than one	
		No	Do Not Have HCP
		Don’t know/Not Sure	
		Refused	Removed for analysis
		Not asked or Missing	
	Delayed Care Because Of Cost	Yes	Care Delayed
		No	Care Not Delayed
		Don’t know/Not sure	Removed for analysis
		Refused	
	Last Medical Checkup	Within past year	Within Last 12 Months
		Within past 2 years	Longer Than 12 Months Ago
		Within past 5 years	
		5 or more years ago	
		Don’t know/Not sure	
		Never	
		Refused	Removed for analysis
Health Specific Variables	Number Permanent Teeth Removed	None	No Teeth
		1 to 5	1 To 5 Teeth
		6 or more, but not all	6 Or More, But Not All Teeth
		All	All Teeth
		Don’t know/Not sure Refused	Removed for analysis
	Self-Defined Health Status	Excellent	Good To Excellent Health
		Very good	
		Good	
		Fair	Fair To Poor Health
		Poor	
		Don’t know/Not Sure	Removed for analysis
		Refused	
	Smoking Status	No	Non-Smoker
		Yes	Current Smoker
		Don’t know/Refused/Missing	Removed for analysis
Access To Dental Care Variable	Dentists Per 100,000 Population Tertile Ranges	Dentists per 100,000 state population ranging from 40.93 to 89.95	40.93–57.23 Per 100,000
			57.24–73.54 Per 100,000
			73.55–89.85 Per 100,000

^a^ Dependent Variable for Bivariate and Logistic Regression Analysis

^b^ Reference Category for Logistic Regression Analysis

For instance, in BRFSS, marital status has nine categories. These were re-coded into three categories: Married, Not Married, and Partner in an Unmarried Couple because these three categories encapsulate the concepts of interest. Likewise, education had nine original categories that were reduced to three through re-coding. The re-coded categories (less than high school, high school graduate, and university graduate) represent the most meaningful ones for this analysis. The dependent variable, timing of last dental visit, had seven possible response categories; were reduced to two: last dental visit longer than 12 months ago/last dental visit within last 12 months. The first of these two categories or factors was used in a multivariable analysis as the dependent variable.

Income was re-coded as it was because the median household income in the US is estimated to be just over $50,000. Our re-code as below $50,000 and $50,000 and higher was the closest approximation given the original categories provided in the BRFSS data analyzed.

Additionally, race/ethnicity was re-coded in the manner it was to avoid the issue of small cell numbers that are problematic for complex samples analyses. Also we re-coded age as we did because the first category 18–44 years takes into account that historically younger adults have been less likely to get annual check-ups regardless of health insurance status, they have also been less likely to have a PCP. While the implementation of the ACA is anticipated to impact access to and utilization of healthcare by younger adults, current research findings are mixed [53]. The ages 45 to 64 were grouped together because the range recognizes middle-aged adults who might be experiencing chronic conditions (e.g., hypertension) and hence health insurance, annual check-ups, having a PCP would all be acutely important. Finally, the range 65 and older was chosen because of Medicare eligibility for that age group.

While rurality has been defined in multiple and not always compatible ways, [54] in this study the geographic locale variable was determined using the metropolitan statistical area (MSA) variable provided in the BRFSS database. MSA is comprised of geographic entities delineated by the Office of Management and Budget (OMB) for use by Federal statistical agencies in collecting, tabulating, and publishing federal statistics [54]. The MSA variable was recoded into the dichotomous categories of rural and metropolitan. Rural residents were defined as people living either within an MSA that had no center city or outside an MSA. Metropolitan residents included all US adults living in a center city of an MSA, outside the center city of an MSA but inside the county containing the center city, or inside a suburban county of an MSA.

The variable active dentists per 100,000 state population was merged with the 2016 BRFSS database. This variable was then recoded into tertiles for analysis. The original data for this variable is collected annually by the American Dental Association [55] and represents an estimate of all active dentists by state. The re-coding was undertaken to organize the data into categories corresponding to high, medium and low access to dental professionals.

All analyses were performed on weighted data as recommended by the CDC. The weighting, calculated by the CDC, uses the most recently available census data to provide a stratified representation of the nation’s non-institutionalized population. All of the performed analyses used the complex samples module available in SPSS. For bivariate analysis, an adjusted F statistic was calculated as the test statistic. For the multivariable analysis performed, an adjusted odds ratio was the calculated test statistic. Only findings from weighted analyses were considered valid. All analyses were performed using SPSS version 25 (IBM, Chicago, IL, USA) with alpha set at < 0.05. The Institutional Review Boards (IRBs) of the researchers’ institutions recognize that the analysis of de-identified, publicly available data does not constitute human subjects research as defined in federal regulations, and as such does not require IRB review. Hence, human subjects review was not sought.

## Results

Table 2 summarizes the study variables and study population weighted frequencies and percentages. For the dependent variable---timing of last dental visit---30.4% of the study population had not had a dental visit in the past 12 months. Also in Table 2, 48.4% of the study population lived in states with the fewest dentists per 100,000 population. Further, while 46.7% of the study population reported having no permanent teeth removed, 7.4% reported having had all of their permanent teeth removed. Additionally, 14.3% reported that they were currently smokers.

**Table 2:** Description of Non-Institutionalized US Adults Included in Oral Health Study 2016 BRFSS Data (weighted *n*=60,512,412)

Variable Category	Study Variables and Factors		Frequency	Percent
Dependent Variable	Last Dentist Visit^a^	Longer Than 12 Months Ago	18,395,902	30.4%
		Within Last 12 Months^b^	42,116,510	69.6%
Demographic Variables	Sex	Male	25,905,063	42.8%
		Female	34,607,349	57.2%
	Age Ranges	18–44 Years	14,512,974	24.0%
		45–64 Years	24,921,543	41.2%
		65 Years And Older	21,077,895	34.8%
	Marital Status	Married	37,034,620	61.2%
		Not Married	21,789,874	36.0%
		Partner In An Unmarried Couple	1,687,918	2.8%
	Geographic Locale	Metropolitan	49,128,575	81.2%
		Rural	11,383,837	18.8%
	Race/Ethnicity	White Non-Hispanic	45,324,018	74.9%
		Black Non-Hispanic	6,427,112	10.6%
		Other Non-Hispanic	3,519,669	5.8%
		Hispanic	5,241,613	8.7%
	Veteran Status	Veteran	7,987,900	13.2%
		Not A Veteran	52,524,511	86.8%
Socio-economic Variables	Education Attained	Less Than High School	6,685,394	11.0%
		High School Graduate	36,053,302	59.6%
		University Graduate	17,773,715	29.4%
	Employment Status	Employed	28,767,825	47.5%
		Not Employed	2,395,899	4.0%
		Not Working By Choice Or Unable To Work	29,348,688	48.5%
	Annual Household Income	Less Than $50,000	29,657,890	49.0%
		$50,000 And More	30,854,522	51.0%
Health Services Variables	Health Insurance Status	Have Health Insurance	56,855,272	94.0%
		Do Not Have Health Insurance	3,657,140	6.0%
	Personal HCP	Have HCP	53,623,164	88.6%
		Do Not Have HCP	6,889,248	11.4%
	Delayed Care Because Of Cost	Care Delayed	5,576,447	9.2%
		Care Not Delayed	54,935,965	90.8%
	Last Medical Checkup	Within Last 12 Months	471,843,067	78.0%
		Longer Than 12 Months Ago	13,328,105	22.0%
Health Specific Variables	Number Permanent Teeth Removed	No Teeth	28,245,450	46.7%
		1 To 5 Teeth	19,294,234	31.9%
		6 Or More, But Not All Teeth	8,480,363	14.0%
		All Teeth	4,492,364	7.4%
	Self-Defined Health Status	Good To Excellent Health	48,713,690	80.5%
		Fair To Poor Health	11,798,722	19.5%
	Smoking Status	Non-Smoker	51,886,829	85.7%
		Current Smoker	8,625,583	14.3%
Access To Dental Care Variable	Dentists Per 100,000 Population Tertile Ranges^c^	40.93–57.23 Per 100,000	29,301,599	48.4%
		57.24–73.54 Per 100,000	16,466,143	27.2%
		73.55–89.85 Per 100,000	14,744,670	24.4%

^a^Dependent Variable for Bivariate and Logistic Regression Analysis

^b^Reference Category for Logistic Regression Analysis

^c^To convert a rate per 100,000 to a percentage move the decimal point three digits to the left

Table 3 displays the results of the bivariate analysis. This analysis yielded that each independent variable and related factors were significantly associated (*p* < 0.05) with the study’s dependent variable. The bivariate analysis was performed to determine which independent variables should be included in the complex samples logistic regression analysis. Since all of the independent variables had a statistically significant relationship with the dependent variable, a decision was made to include all of the independent variables in the multivariable logistic regression model tested.

**Table 3:** Complex Samples: Bivariate Analysis Last Dental Visit by Study Covariates 2016 BRFSS Data

Variable Category	Covariates and Factors		Last Dentist Visit		*p*-value*
			Longer Than 12 Months Ago	Within Last 12 Months	
Demographic Variables	Sex	Male	46,602,666 / 52.7%	76,940,221 / 46.5%	.000
		Female	41,854,895 / 47.3%	88,495,464 / 53.5%	
	Age Ranges	18–44 Years	42,687,478 / 48.2%	75,122,926 / 45.4%	.000
		45–64 Years	28,494,110 / 32.2%	56,817,450 / 34.3%	
		65 Years And Older	17,291,479 / 19.5%	33,508,777 / 20.3%	
	Marital Status	Married	37,154,844 / 42.3%	90,321,846 / 55.0%	.000
		Not Married	45,303,410 / 51.5%	67,051,356 / 40.8%	
		Partner In An Unmarried Coupled	5,450,378 / 6.2%	6,851,984 / 4.2%	
	Geographic Locale	Metropolitan	18,962,965 / 77.1%	45,517,333 / 83.4%	.000
		Rural	5,625,139 / 22.9%	9,036,397 / 16.6%	
	Race and Ethnicity	White Non-Hispanic	49,433,721 / 57.0%	107,745,288 / 66.3%	.000
		Black Non-Hispanic	12,121,155 / 14.0%	17,291,109 / 10.6%	
		Other Non-Hispanic	6,872,299 / 7.9%	13,582,687 / 8.4%	
		Hispanic	18,224,520 / 21.0%	23,895,023 / 14.7%	
	Veteran Status	Veteran	8,762,047 / 9.9%	18,220,075 / 11.0%	.000
		Not A Veteran	79,426,563 / 90.1%	146,770,809 / 89.0%	
Socio-economic Variables	Education Attained	Less Than HS	19,851,625 / 22.6%	15,425,520 / 9.4%	.000
		HS Graduate	54,196,392 / 61.6%	95,552,211 / 58.0%	
		University Graduate	13,978,080 / 15.9%	53,802,139 / 32.7%	
	Employment Status	Employed	46,559,563 / 52.6%	96,839,752 / 58.5%	.000
		Not Employed	6,439,077 / 7.3%	7,109,706 / 4.3%	
		Not Working By Choice Or Unable To Work	35,449,462 / 40.1%	61,454,059 / 37.2%	
	Annual Household Income	Less Than $50,000	50,843,550 / 69.7%	59,608,418 / 43.1%	.000
		$50,000 And More	22,098,057 / 30.3%	78,628,095 / 56.9%	
Health Services Variables	Health Insurance Status	Have Health Insurance	70,404,914 / 80.1%	152,925,843 / 92.9%	.000
		Do Not Have Health Insurance	17,458,226 / 19.9%	11,647,884 / 7.1%	
	Personal HCP	Have HCP	59,975,447 / 67.9%	137,436,415 / 83.2%	.000
		Do Not Have HCP	28,336,795 / 32.1%	27,810,053 / 16.8%	
	Delayed Care Because Of Cost	Care Delayed	18,110,419 / 20.6%	14,939,460 / 9.0%	.000
		Care Not Delayed	70,017,530 / 79.4%	150,164,890 / 91.0%	
	Last Medical Checkup	Within Last 12 Months	52,110,157 / 59.0%	126,703,017 / 76.7%	.000
		Longer Than 12 Months Ago	36,246,201 / 41.0%	38,568,615 / 23.3%	
Health Specific Variables	Number Permanent Teeth Removed	No Teeth	42,866,454 / 49.5%	96,478,778 / 59.2%	.000
		1 To 5 Teeth	23,915,004 / 27.6%	48,736,369 / 29.9%	
		6 Or More, But Not All Teeth	10,663,744 / 12.3%	14,325,222 / 8.8%	
		All Teeth	9,108,344 / 10.5%	3,301,783 / 2.0%	
	Self-Defined Health Status	Good To Excellent Health	64,961,197 / 73.7%	142,814,027 / 86.5%	.000
		Fair To Poor Health	23,137,576 / 26.3%	22,321,669 / 13.5%	
	Smoking Status	Non-Smoker	63,410,623 / 75.8%	138,254,361 / 87.9%	.000
		Current Smoker	20,248,194 / 24.2%	19,040,890 / 12.1%	
Access To Dental Care Variable	Dentists Per 100,000 Population Tertile Ranges	40.93–57.23 Per 100,000	47,857,926 / 54.7%	77,433,898 / 47.4%	.000
		57.24–73.54 Per 100,000	19,880,342 / 22.7%	42,160,943 / 25.8%	
		73.55–89.85 Per 100,000	19,771,381 / 22.6%	43,828,997 / 26.8%	

*Significance is based on the adjusted F and its degrees of freedom

Table 4 displays the results of the complex samples logistic regression analysis performed using *last dental visit longer than 12 months ago* as the dependent variable. Seventeen independent variables constituting 26 factors were included in the analysis. Of the demographic variables included in the logistic regression analysis, three (sex, geographic locale, race/ethnicity) yielded significant results for one or more factors. Males, when compared to females, had greater odds of not having had a dental visit in the past 12 months (OR 1.370, CI 1.240–1.455) as did adults living in a rural area (OR 1.095, CI 1.034–1.160) when compared to those living in a metropolitan one. Analysis of the race/ethnicity factors indicated that Non-Hispanic Blacks (OR 1.296, CI 1.187–1.416) and Non-Hispanic Other races (OR 1.155, CI 1.011–1.320) had significantly greater odds of not having visited a dentist in the last 12 months when compared to Non-Hispanic Whites.

**Table 4:** Complex Samples Logistic Regression Analysis: Last Dental Visit Longer than 12 Months Ago as Dependent Variable 2016 BRFSS Data

Variable Category	Covariates	Factors	Adjusted Odds Ratio	95% Confidence Interval	
				Lower	Upper
Demographic Variables	Sex	Male vs. Female	1.370	1.290	1.455
	Age Ranges	18–44 Years vs. 65 Years And Older	1.090	.997	1.191
		45–64 Years vs. 65 Years And Older	1.050	.982	1.123
	Marital Status	Married vs. Partner In An Unmarried Coupled	.878	.724	1.066
		Not Married vs. Partner In An Unmarried Coupled	.964	.793	1.170
	Geographic Locale	Rural vs. Metropolitan	1.095	1.034	1.160
	Race And Ethnicity	Black Non-Hispanic vs. White Non-Hispanic	1.296	1.187	1.416
		Other Non-Hispanic vs. White Non-Hispanic	1.155	1.011	1.320
		Hispanic vs. White Non-Hispanic	1.051	.929	1.188
	Veteran Status	Veteran vs. Not A Veteran	.945	.873	1.024
Socio-economic Variables	Education Attained	Less Than High School vs. University Graduate	2.228	2.004	2.477
		HS Graduate vs. University Graduate	1.555	1.467	1.648
	Employment Status	Not Employed vs. Employed	1.174	1.027	1.343
		Not Working By Choice Or Unable To Work vs. Employed	1.021	.955	1.092
	Annual Household Income	Less Than $50,000 vs. $50,000 And More	1.992	1.871	2.120
Health Services Variables	Health Insurance Status	Do Not Have Health Insurance vs. Have Health Insurance	1.317	1.162	1.491
	Personal HCP	Do Not Have HCP vs. Have HCP	1.396	1.279	1.525
	Delayed Care Because Of Cost	Care Delayed vs. Care Not Delayed	1.568	1.428	1.721
	Last Medical Checkup	Longer Than 12 Months Ago vs. Within Last 12 Months	1.877	1.756	2.005
Health Specific Variables	Self-Defined Health Status	Fair to Poor Health vs. Good to Excellent Health	1.523	1.427	1.626
	Number Permanent Teeth Removed	1 To 5 Teeth Vs. No Teeth	.991	.932	1.055
		6 Or More, But Not All Teeth Vs. No Teeth	1.390	1.286	1.502
		All Teeth Vs. No Teeth	6.331	5.721	7.007
	Smoking Status	Current Smoker vs. Non-Smoker	1.472	1.373	1.578
Access To Dental Care Variable	Dentists Per 100,000 Population Tertile Ranges	40.93–57.23 Per 100,000 vs. 73.55–89.85 Per 100,000	1.185	1.103	1.274
		57.24–73.54 Per 100,000 vs. 73.55–89.85 Per 100,000	.999	.924	1.080

All of the socioeconomic variables (education attained, employment status, annual household income) included in the logistic regression analysis yielded significant results for one or more factors with adults with less than a high school education (OR 2.28, CI 2.004–2.477) as well as those with only a high school education (OR 1.555, CI 1.467–1.648) having greater odds of not having seen a dentist in the past 12 months when compared to adults who were university graduates. Additionally, US adults who reported being unemployed (OR 1.174, CI 1.027–1.343), in contrast to employed, and/or having an annual household income of less than $50,000 (OR 1.992, CI 1.871–2.120), rather than $50,000 and higher, had greater odds of not having had a dental visit in the last 12 months.

All four health services deficits variables (last routine medical exam, have personal healthcare provider, health insurance status, and/or delayed medical care because of cost) included in the logistic regression analysis showed significant results. US adults without health insurance (OR 1.317, CI 1.162–1.491) rather than with health insurance, without a personal HCP (OR 1.396, CI 1.279–1.525) rather than with a HCP, who had delayed medical care because of cost (OR 1.568, CI 1.428–1.721) rather than not deferring cast because of cost, and who had their last routine medical visit longer than 12 months ago (OR 1.877, CI 1.756–2.005) rather than within the last 12 months had greater odds of not having a dental visit within the last 12 months.

Of the three health variables included in the complex samples logistic regression analysis (self-defined health status, number of permanent teeth removed, and smoking status), all yielded significant results. US adults who defined their health status as fair to poor (OR 1.523, CI 1.427–1.626) rather than good to excellent, had six or more (OR 1.380, CI 1.286–1.502) and/or all of their permanent teeth (OR 6.331, CI 5.721–7.00) removed rather than none of their teeth removed, and were current smokers (OR 1.472, CI 1.373–1.578) rather than non-smokers had greater odds of not having had a dental visit in the past 12 months. Finally, US adults living in states with the fewest number of active dentists per 100,000 population (OR 1.185, CI 1.103–1.274) rather than the highest number of active dentists per 100,000 population had greater odds of not having had a dental visit in the past 12 months.

## Discussion

Evidence supports the connection between oral health and overall health [6–14] and an understanding of the risk factors associated with oral health disparities provides insight about the health service needs within a population. To gain a better understanding of these risk factors, this study explored the associations between HSDs and not visiting a dental professional in the past 12 months. Enhancing the utilization of dental services is a fundamental step for addressing oral health disparities in the US.

A key finding of this analysis is that one-third of US adults had not seen a dentist within the past 12 months, which despite HP2020’s emphasis on oral health is a level similar to previously reported levels (approximately 32%) [56]. In contrast, 22.0% of US adults had not had a routine medical checkup within the past 12 months (a 36.4%difference from the proportion of US adults seeing a dental professional in the past 12 months). Since more adults visit a physician annually than a dental professional, this suggests that a more robust integration of dental with medical services might increase access to and utilization of dental care. Such an integration could increase bi-directional referrals and better coordination of necessary care (both primary care and dental care) [1–4]. For example, a dentist may be asked by a patient to address an esthetic issue yet have signs and symptoms of an uncontrolled chronic illness that merits referral to a medical practitioner. While both the American Dental Association and American Medical Association have referral guidelines within their disciplines, neither publishes guidelines for integrating dental and medical care [57].

Our findings revealed that all four of the HSD variables examined---no health insurance, no personal HCP, delaying healthcare because of cost, and last routine medical visit longer than 12 months ago---were significantly associated with not visiting a dental professional in the last 12 months. These findings suggest that strategies to integrate dental care and medical care *which address* HSDs have the potential to improve overall health. If nothing else, inclusive medical insurance coverage must embrace oral health. These insurance plans should be affordable (to avoid delaying care because of cost) and mandate care coordination in order to truly support comprehensive healthcare. At present an estimated 67% of working age US adults have some form of dental insurance while 83% of same age adults have health insurance. Furthermore, the burden of unaddressed dental disease falls, for the most part, on the medical and not dental care system [58].

In addition to HSDs, this study updated risk factors and identified several other.

potential areas of focus to improve dental care utilization. Populations with a higher socioeconomic burden, racial minorities, males, and those with less education all had greater odds of not having seen a dentist in the past 12 months. Hence, similar to all healthcare, improving/increasing access to dental care requires at the minimum taking socioeconomic factors into account. Presently, dental insurance limitations remain a persistent deterrent to preventive and restorative dental care [59]. The ACA attempts to tackle these socioeconomic barriers. However, despite Medicaid expansion, the ACA impact on oral health has been small [60–63]. As currently constructed, the ACA only modestly increased dental care utilization for children [32] and does not appear to have had a substantial impact on dental care among adults [64]. This is not surprising since there is no adult dental insurance requirement in the ACA and Medicaid payments for dental care are often low. While strengthening and utilizing the ACA as a tool to coordinate dental and medical services with enhanced dental coverage represents one strategy to improve the quality and efficiency of overall healthcare (health and dental), the current political climate could lead to any number of possibilities from repeal to reform. Since the current state of public opinion favors maintaining and reforming the ACA [65], this moment in history might present an opportunity to address the importance of oral health in a meaningful way.

Rural residency emerged as an independent factor associated with lower dental care utilization even after controlling for HSDs, socioeconomic factors, demographics, and dentist supply. While an earlier study looking at a single state [66] did not associate rurality with dental care utilization, our results suggest that factors unique to a rural setting might play a role in dental care utilization and that rural residents may benefit from programs targeted at rural settings. Other researchers found that rural residents are more likely than their non-rural counterparts to delay dental care until being in pain, [67] suggesting a potential benefit from educational programs targeted at the importance of preventive care and early intervention. Addressing rural needs is especially important since rural adults are more likely to have untreated dental disease and poorer oral health. In rural American there are more physicians per capita then dentists [68] and Khan, et al. [69] recommended enhancing the integration of rural oral healthcare into primary care as a strategy to mitigate the challenges of providing dental care in rural settings.

The study results also revealed that those with no teeth or fewer teeth were far less likely to seek dental care. Tooth loss or being edentulous increases the risk of oral cancer by 2 to 3 fold even after controlling for smoking and alcohol use [70]. Failure to have regular dental care might be a missed opportunity for detecting cancer in an earlier stage which improves outcomes [71]. In addition, routine dental care helps preserve existing teeth which is associated with better health outcomes. Our finding suggests that establishing a recommendation for dental assessment as part of routine medical care for those with teeth loss offers the potential for improving the health of these individuals.

This study does have several limitations. One limitation to consider in interpreting the data is the absence of information on the purpose of the dental visit. Regularly scheduled maintenance provides oral disease prevention and early detection. Dental emergencies however, for treatment of acute pain do not allow opportunity for preventive treatment or hygiene. Risk factors leading to emergency department or other emergency visits require further investigation. A second limitation is that possible explanations for utilization differences in dental care such as attitudes to care are not captured by the BRFSS survey. Another limitation is that BRFSS data is self-recall data and hence subject to recall bias and also social desirability bias, that is if a respondent believes that one should see a dentist annually they might report a dental visit even if one does not occur. Furthermore, the variable used in the analysis for access to dentists---*active dentists per 100,000 state population*---does not take into account factors such as number of dentists within a state who do not accept Medicaid or the geographic distribution of dentists in a state. Finally, the race/ethnicity variable does not have a distinct American Indian/Alaska Native category. This population designation is merged into the non-Hispanic Other race/ethnicity category.

## Conclusions

HSDs are an independent risk factor strongly associated with lower rates of dental care utilization. Any strategy to enhance the integration of medicine and dentistry should take HSDs into account. In addition, despite national goals for achieving health equity, persistent disparities in dental utilization remain for several demographic groups. Future research about, enhancing interdisciplinary collaboration and strategies targeting interventions for at risk individuals offers the promise of improved oral health.

## Abbreviations

ACA: Affordable Care Act
BRFSS: Behavioral Risk Factor Surveillance System survey
CDC: Centers for Disease Control and Prevention
FQHCs: Federally Qualified Health Centers
HCP: healthcare provider
HP2020: Healthy People 2020
HSDs: Health service deficits
MSA: metropolitan statistical area
SPSS: Statistical Package for Social Scientists
US: United States
WHO: World Health Organization
